# Organizational Network Analysis of SAMHSA’s Technology Transfer Center (TTC) Network

**DOI:** 10.1007/s11414-023-09867-y

**Published:** 2023-10-23

**Authors:** Jon Agley, Ruth Gassman, Kaitlyn Reho, Jeffrey Roberts, Susan K. R. Heil, Lilian Golzarri-Arroyo, Kate Eddens

**Affiliations:** 1 Prevention Insights, Department of Applied Health Science, School of Public Health Bloomington, Indiana University Bloomington, 809 E. 9th St, Bloomington, IN 47405 USA; 2 American Institutes for Research (AIR), Arlington, VA USA; 3 Biostatistics Consulting Center, School of Public Health Bloomington, Indiana University Bloomington, Bloomington, IN USA; 4 Department of Epidemiology and Biostatistics, School of Public Health Bloomington, Indiana University Bloomington, Bloomington, IN USA

**Keywords:** TTC, Technology transfer center, SAMHSA, Network analysis, Evaluation, Addiction, Prevention, Mental health

## Abstract

Technology transfer centers (TTCs) facilitate the movement of evidence-based practices in behavioral healthcare from theory to practice. One of the largest such networks is the Substance Abuse and Mental Health Services Administration’s (SAMHSA) TTC Network. This brief report shares findings from an organizational network analysis (ONA) of the network conducted as part of an external evaluation. For non-supervisory TTCs (*n* = 36) across three focus areas (addiction, prevention, and mental health), the authors computed network density, harmonic closeness, and non-null dyadic reciprocity for five types of interactions (e.g., “collaborated in workgroups”), then, for each interaction type, used Welch’s *T*-test to compare mean harmonic closeness of standalone TTC grantees versus multiple-TTC grantees. ONA identified potentially isolated regional TTCs as well as mismatches between some centers’ desired scope and their network centrality and enabled investigation of broader questions around behavioral health support systems. The approach appears useful for evaluating TTCs and similar support networks.

## Introduction

In most areas of healthcare, novel findings, innovations, and evidence-based practices do not naturally or automatically transition from research to practice. Rather, they must be distilled and intentionally moved into practice systems where they can be implemented, and people must be taught to use them^1^. Falling broadly under the umbrella of dissemination and implementation science^2^, the support systems that facilitate this movement and training (e.g., capacity-building) are sometimes labeled technical assistance (TA) centers^3^ or technology transfer centers (TTCs)^4, 5^.

One of the largest such systems currently operating in the USA is the Substance Abuse and Mental Health Services Administration’s (SAMHSA’s) TTC Network, which is composed of 39 centers covering addiction treatment (ATTC), prevention (PTTC), and mental health (MHTTC), with a combined grant-cycle (5-year) budget of approximately US $130 million. The ATTC Network was established in 1993, while the PTTC and MHTTC Networks were established in 2018. Domestically, each network is composed of 10 regional centers aligned with geographic regions of the USA, two national focus area centers (NFACs) focused on American Indian and Alaska Native health and Hispanic/Latino health, respectively, and one national coordinating office (NCO)^6^. Despite the network’s relative newness, important research on SAMHSA’s TTCs has been published^7–10^ (and studies from the standalone ATTC Network were also published prior to 2018^11^). However, this work has typically originated from inside the network and/or has been broader but focused on changes to TA resulting from COVID-19. Given the substantial national investment in, and scope of, the TTC Network, it is critical that it be rigorously evaluated^12^ from a broader, external, and independent perspective.

In 2019, the authors initiated a large-scale, multi-part, 2-year formative evaluation of SAMHSA’s TTC Network. Because the TTCs are not independent from each other, but rather work as parts of a formal network, one component of the evaluation was a sociocentric (“whole network”) organizational network analysis (ONA)^13^ of the TTC Network to examine group dynamics and the overall group structure^14^. This ONA evaluation was distinct from individual-level evaluations because it focused on relationships of the TTCs within the network^15^. The authors examined three different evaluation questions separately for five different types of interactions, such as “collaborated in a workgroup.”^16^ The evaluation also addressed a fourth question that was unrelated to interaction type by using egocentric networks in which TTCs identified their closest external “network neighbors.”^13^ The evaluation questions were as follows:

1. How interconnected is the TTC Network for each interaction type?
2. Which TTCs are most interconnected in each interaction network?
3. Is awarding multiple TTC contracts to the same recipient (e.g., university) associated with TTC Network centrality for any interaction type?
4. Are any specific external entities independently collaborating with multiple TTCs?

## Methods

Direct invitations to the organizational network survey were sent to the project director (or co-director) at each TTC, who was asked to complete a survey on QualtricsXM (a copy of the survey is available as Supplement 1). TTC representatives responded from March 12, 2020, through April 10, 2020. Individuals who served as project directors for multiple TTCs were asked to complete a survey for each TTC they directed. While data on NCOs were collected, they were excluded from the analysis because NCOs’ unique supervisory roles within the overall TTC Network meant that their interactions would not appropriately be aggregated with those of the other sites. These study procedures were not considered human subjects research because the focus was on organizations (IRB #1910589313). To preserve the confidentiality of TTCs, designations (Regions 1 through 10 and NFACs) have been randomized to letters A through L in the manuscript as well as in the analytic code and data.

### Procedures

Each respondent was asked, “Thinking only about the past three (3) months, with which other TTCs have you directly interacted (e.g., collaboration, providing or receiving support)?” The question also noted, “If the only interaction with another TTC was shared attendance (e.g., both entities were on the same Zoom call) then you should not include that TTC.” TTCs were then presented with a list of all other TTCs across types and networks (including NFACs). After TTCs selected all the other TTCs with which they had interacted using checkboxes, that list was drawn forward to a new question, which asked: “For each TTC you selected, please indicate the types of interactions you have had (select all that apply).” The available interaction types were collaborated on product development, collaborated on training delivery, collaborated in a workgroup, provided in-kind support, received in-kind support, and other.

For Aim 4, each TTC was asked, “With what organizations and individuals outside the TTC network has your TTC had significant interactions in the past three (3) months? We know TTCs interact with many organizations—but we are only interested in up to 10 relationships that your TTC considers to be most important to its work as a TTC. This can include organizations to which the TTC provides services, as well as organizations from which the TTC receives services or support, as well as any other partners you believe to be meaningful to your TTC’s work.”

### Analysis parameters

All analyses were conducted using RStudio 1.3.1093 (R 4.0.2) with the “sna” package. Analytic code and associated datasets are provided in Supplement 2. The authors generated one visual map for each interaction type (see Supplement 3). Since the analyses examined relationships among TTCs (the elements of interest), each TTC was represented by a “vertex.” For collaboration variables, analysts treated assertion by *either* of a pair of TTCs that collaboration existed as a relationship, but still measured whether such assertions were reciprocal.

Our primary metrics of interest for each interaction type were as follows:

- Density, which is the number of direct connections (“edges”) that exist in a network relative to the number of edges that would exist if every vertex were connected to every other vertex.
- Closeness, which describes the average path length to all other vertices. For this study, we used “harmonic closeness,” which is the ratio of how centralized a vertex is relative to how centralized it could possibly be, represented as a percentile from 0 to 100%. The higher the percentile, the more interconnected the vertex is within that map.
- Reciprocity (collaborations only), in this case, “non-null, dyadic reciprocity,” which is the proportion of pairs of vertices that *mutually* indicate that they share an edge divided by the proportion of pairs for which at least one vertex indicates it shares an edge.

The evaluators also examined whether closeness of a TTC within the network was associated with whether it was a standalone grantee or a grantee with multiple (two or three) separate TTC awards. Information about TTC center funding was derived from documentation provided by SAMHSA. Those comparisons used Welch’s two-sample *T*-test,^17^ which does not carry the assumption of equality of variances and adjusts degrees of freedom at the analytic stage.

## Results

All 36 TTCs participated in the study. No data were missing, but one TTC (MHTTC I) selected all other TTCs (e.g., 35) for “collaboration in workgroups,” which was a substantial outlier (the next three highest values were 13, 10, and 10), so MHTTC I was excluded from that analysis. Visually (see Supplement 3), the authors observed that the network clustering for the interaction types with non-directional ties supported the validity of the analytic approach because the TTC types (ATTC, PTTC, MHTTC) tended to cluster together even though no information about TTC type was included in the modeling. Composite measures and statistical tests are shown in Table 1.

**Table 1:** Network analysis and *T*-test results

INTERACTION TYPE	MEAN HARMONIC CLOSENESS (STANDALONE)	MEAN HARMONIC CLOSENESS (MULTIPLE GRANTEEs)	DENSITY (%)	RECIPROCITY (%)	*T*-TEST OF CLOSENESS
COLLABORATION ON PRODUCT DEVELOPMENT	48.3%	54.6%	17.5%	21.8%	*t* = − 2.933, *df* = 24.19, *p* = 0.007
COLLABORATION ON TRAINING	51.3%	55.4%	18.1%	28.1%	*t* = − 1.550 *df* = 29.02 *p* = 0.132
COLLABORATION IN WORKGROUPS	52.9%	56.3%	19.8%	32.2%	*t* = − 1.551 *df* = 27.34 *p* = 0.132
PROVIDED IN-KIND SUPPORT	23.6%	20.9%	3.8%	-	*t* = 0.553 *df* = 32.50 *p* = 0.584
RECEIVED IN-KIND SUPPORT	14.1%	15.4%	3.1%	-	*t* = − 0.345 *df* = 33.61 *p* = 0.732
INTERACTION TYPE	Highest harmonic closeness				
COLLABORATION ON PRODUCT DEVELOPMENT	MHTTC I, ATTC J, PTTC G, and ATTC K				
COLLABORATION ON TRAINING	ATTC C, MHTTC L, ATTC G, and PTTC G				
COLLABORATION IN WORKGROUPS	MHTTC L, PTTC L, ATTC J, and PTTC G				
PROVIDED IN-KIND SUPPORT	MHTTC B, MHTTC G, PTTC G, and MHTTC D				
RECEIVED IN-KIND SUPPORT	MHTTC G, MHTTC B, ATTC G, and ATTC D				

### Aim 1

Density values indicated just under 1/5 of collaborations existed in the network out of all theoretically possible collaborations (e.g., if each TTC collaborated with all 35 others). For providing or receiving in-kind support, which are directional links, TTCs were far less connected (density under 4% for both).

### Aim 2

The TTCs with the highest harmonic closeness varied by interaction type, with some TTCs being highly centralized for more than one type of interaction. The four most centralized TTCs for each interaction type are noted in Table 1. Region G TTCs were particularly central, with at least one TTC among the most centralized for each interaction type. In contrast, Region F TTCs tended to be more isolated than other TTCs for each interaction type (not shown in Table 1, see Supplement 3).

### Aim 3

The analyses provide some evidence to suggest that awarding multiple TTC contracts to the same recipient may be associated with increased network centralization for collaboration on product development (*t* =  − 2.933, *df* = 24.19, *p* = 0.007), but may not be associated with other interaction types.

### Aim 4

The evaluators expected to be able to generate “egocentric” network maps showing how entities outside of the TTC Network collaborated with multiple TTCs. However, 319 different entities were listed as collaborators (out of a theoretically possible 360, but not all TTCs listed 10 entities), with almost no overlap (< 1%). Such data do not support the inference that any external entity was interlinked with many different TTCs to do important or substantive work.

## Discussion

Researchers within the TTC Network have published multiple studies around TTC processes and outcomes, including the works previously cited^7–10^ as well as recent multi-year evaluations^18^, case study series^19^, and other analyses^20^. This work has generally been conducted *within* one of the three TTC Networks. However, possibly because funding for evaluation by any given TTC is contractually restricted, cross-network evaluation studies, including sociocentric network analyses of the type conducted in this study, have not previously been conducted.

The ONA component of our team’s evaluation affirmed that this approach to evaluating agency interactions and collaboration could be used to assess TA/TTC Network structures. ONA studies in public health are not especially rare^21–23^. However, findings from sociocentric analyses can sometimes be limited by the degree to which organizations choose to participate in data collection^21^. In contrast, SAMHSA’s TTC Network seemed particularly well-suited for sociocentric ONA, as the closed nature of the network (e.g., a specific, funded set of TTCs) and formal nature of the evaluation (e.g., responses from directors were expected by SAMHSA) further ensured the authors’ ability to collect a complete dataset. Similar analyses could likely be repeated in future evaluations and/or with subsequent TTC cohorts.

The ONA analyses also produced actionable information for SAMHSA to use in supporting its TTCs. First, the analyses showed fairly high levels of collaboration between TTCs within and across networks and regions. Importantly, the ONA allowed the evaluators to identify a particular region (Region F) where all three TTCs were relatively isolated for more than one interaction type, with ATTC F being isolated for three interaction types, and to recommend support and outreach to that region. Second, the premise of the NFACs is that they conduct cross-cutting work that is not limited to certain regions^6^, yet harmonic closeness data did not suggest that NFACs were consistently among the most centralized, although selected NFACs were among the more central TTCs for some interaction types. Those findings allowed the authors to suggest reexamination of the NFAC structure to learn why they were not more central to the collaboration and support networks and to build in additional qualitative evaluation components related to NFACs later in the overall evaluation. In both cases, ONA enabled potentially useful feedback to be delivered to SAMHSA that may not have been readily identifiable using other types of formative evaluation.

### Limitations

The most substantive limitation in interpreting these data is the low level of reciprocity in the collaboration networks. Reciprocity does not measure the actual collaborations between the TTCs, but rather the degree to which TTCs mutually asserted the same collaborations. If this discrepancy resulted from problems with memory or recall, then it may simply demonstrate data triangulation (e.g., multiple sources to identify single collaborations). On the other hand, if these differences resulted from different “thresholds” of what constitutes collaboration, then the analyses are more likely to reflect a “lowest threshold standard” (e.g., even “weak” collaborations are being documented). Furthermore, in the latter case, TTCs operating at the same recipient site might be more likely to indicate collaborations with each other. It is also theoretically possible that the reciprocity value for some dyads was suppressed in the specific case where a dyad of TTCs took the survey several weeks apart from one another, and their only interaction was in a period not covered by the overlapping retrospective timeframe provided in the question. While we cannot know whether that occurred, since all TTCs took the survey within the same 30-day period (and most within the same 21-day period), we view it as somewhat unlikely. Finally, it is also possible that the data were subject to social desirability bias.^24^ TTCs were aware that they were being evaluated and may have responded in a way that they thought SAMHSA or the evaluators would want to see. As with all studies, these outcomes and conclusions should be interpreted cautiously and not used to make broad inferences or decisions without consideration of the totality of available evidence.

## Implications for Behavioral Health

As previously noted, this work is a component of the first independent evaluation of SAMHSA’s new national TTC Network. In that context, these findings may help support the design and content of subsequent TTC funding opportunity announcements and evaluations of the network, in addition to the direct utility for SAMHSA described in the “Discussion.” More generally, the findings facilitate discussion of two specific nuances of behavioral health networks in the USA that extend beyond the TTC Network.

First, though not formally published or documented, academic “water cooler” discussion around the funding of behavioral health centers sometimes suggests that having multiple co-located centers can reduce external collaboration. The evaluators investigated this possibility but did not observe significant differences between mean harmonic closeness and whether TTCs were single-grant or multi-grant sites. The exception was “collaboration on product development,” where multi-grant sites were somewhat *more* central to the network than single-grant sites (6.3% higher mean harmonic closeness). This finding might tentatively suggest that agency network interactivity may not be hampered by issuing multiple TA/TTC contracts to the same recipients.

Second, because there are many different types of networks related to behavioral health work in the USA, the authors also expected to find an interlinked egocentric network among TTCs where specific external entities were working independently with multiple TTC sites across regions and focus areas. However, responses to this survey item were so dispersed that it was not even possible to construct a visual network map. In particular, TTCs tended to interact with localized subcontractors and local- and state-level organizations representing their core audiences, including those that might be involved with helping to identify needed types of TA (separately, stakeholder involvement with TTC decision-making was identified through components of the evaluation that have not yet been published). This is not to claim that larger partnerships potentially involving TTCs are not potentially forthcoming. For example, in 2020, a framework was published that, in part, describes possible behavioral health-based collaborations between land-grant university cooperative extensions and the SAMHSA TTC Network.^25^

## Data Availability

De-identified data from this study, the analytic code used to conduct the analyses, and the materials (i.e., survey) used to conduct this study are available as supplemental files (Supplements 1 through 3) on the open science platform (https://osf.io/sjak4/?view_only=7d067f744dab47ff936f83892421573f).
